# Vaginal Colonization by *papG* Allele II^+^ *Escherichia coli* Isolates from Pregnant and Nonpregnant Women as Predisposing Factor to Pyelonephritis

**DOI:** 10.1155/2013/860402

**Published:** 2013-06-19

**Authors:** Sareaa Maseer Gatya Al-Mayahie

**Affiliations:** Department of Biology, College of Science, University of Wasit, Al-Kut, Wasit Province, Iraq

## Abstract

Vaginal (61) and fecal (61) *Escherichia coli* isolates from pregnant and nonpregnant women (18–45 years old) were surveyed for *papG* alleles by PCR technique. *papG* allele II was the most prevalent among both vaginal (32.7%) and fecal (3.2%) isolates, whereas other alleles were found only among vaginal isolates (1.6% for alleles I and III and 3.2% for alleles II + III). *papG* ^+^ pregnant women's isolates did not differ significantly from those of nonpregnant in possession of *papG* allele II (90% versus 73.3%), whereas both (32.7%) differed significantly (*P* ≤ 0.05) in comparison with fecal isolates (3.2%). The vast majority of *papG* allele II^+^ vaginal isolates were clustered in group B2 (81.8%) and much less in group D (18.1%). Also, most of them were positive for *fimH* (100%), *papC* (100%), *iucC* (90.9%), and *hly* (72.7%), and about half of them were positive for *sfa/foc* (45.4%). In addition, the mean of VFs' gene possession was 3.5 (range from 2 to 5). It can be concluded that vaginal colonization by *papG* allele II^+^ *E. coli* is possibly one of the predisposing factors of both pregnant and nonpregnant women to pyelonephritis, but its potential may be modified by other factors especially host factors.

## 1. Introduction

Bacterial adherence is an essential step in all infections which involves surface interactions between specific receptors on the mammalian cell membrane and ligands on the bacterial surface. Tissue specificity of infection is determined significantly by the presence or absence of specific receptors on mammalian cells [1]. The ability of uropathogenic *Escherichia coli* (UPEC) to adhere to host uroepithelia is an important stage in the successful colonization of the urinary tract and pathogenesis of urinary tract infection (UTI). The principal adherence organelle of UPEC is P fimbriae, which mediates Gal(*α*1-4)Gal-specific binding via the adhesin molecule PapG [2]. The three molecular variants (I to III) of the adhesin are coded by the adhesin gene *papG *of which there are three known alleles [3]. These variants exhibit different receptor binding specificities [4]. Naturally, *papG* alleles occurin four combinations, that is, class *Ι* plus III, class III only, class II plus III, and class II only [2, 5]. According to the receptor specificity of the PapG adhesin, p-fimbriated uropathogenic *E. coli* is clinically divided into two subtypes: *papG* allele II^+^ strains associated with pyelonephritis and bacteremia, and *papG* allele III^+^ strains associated with cystitis but have been found in pyelonephritis and bacteremia [2, 5–7]. 

The most common extraintestinal *E. coli* infection in healthy women is UTIs [8, 9] which develop in an ascending manner, with *E. coli *gaining access to the bladder via the urethra, and the initial colonization of the vaginal mucosa is considered a critical step toward infection [10–12]. These colonizers of the female introitus predispose the women to recurrent UTI [13]. Acute cystitis is extremely common among reproductive-age women, whereas acute pyelonephritis, while much less common, is associated with high per-episode costs and morbidity [14] and is more common in pregnant women than in nonpregnant women [11]. As vaginal colonization by UPEC is a possible previous step to urinary tract infection, this work was designed to see if there is any difference in *papG* alleles' distribution (especially *papG* allele II) among *E. coli* vaginal isolates from pregnant and nonpregnant women and also to evaluate the possible ability of *papG* allele II^+^ isolates to cause pyelonephritis by genotypic analyses of* E. coli* phylogenetic groups and extraintestinal pathogenic *E. coli* virulence factors (ExPEC VFs). Fecal isolates from pregnant and nonpregnant women were included for comparison.

## 2. Materials and Methods 

### 2.1. Bacterial Isolates

 This study included 122 *E. coli* isolates (61 vaginal and 61 fecal isolates). Vaginal isolates (23 from pregnant and 38 from nonpregnant women) were recovered as significant growth from high vaginal swabs collected by gynecologists from pregnant and nonpregnant women (aged 18–45 years) clinically diagnosed as having symptomatic genital tract infection, without investigating the exact cause of infection (women with vaginal discomfort, causes of which had not been clarified by gynecological examination). The swabs were streaked immediately after collection on eosine methylene blue agar (EMB) (Himedia) and blood agar plates. The plates were incubated at 37°C for 24–48 hours at ambient air. 

 Fecal isolates (included for comparison) were recovered from healthy volunteers (pregnant (30 isolates) and nonpregnant (31 isolates) women, aged 18–45 years). A single fecal specimen was collected per person. The specimens were processed according to Plos et al. [15] by dilution streaking the fecal material onto EMB. After incubation, from each plate the last three colonies (with the appropriate color and morphology, that is, characteristics of *E. coli*) at the end of the streak area were selected and subcultured onto EMB plate again, incubated, subcultured again onto tryptic soy agar plates (TSA) (Himedia), and then kept in the refrigerator for further work. 

 All this study-included isolates were collected over a 2-year period from May 2008 to June 2010 at Obstetrics and Gynecology Clinics in Al-Kut/Wasit Province/Iraq, and were identified by conventional biochemical tests [16, 17].

### 2.2. Detection of *papG *Alleles by PCR Technique

All isolates were screened for the presence of the three *papG* alleles (I, II, and III) by a multiplex PCR assay using specific primers (Table 1). For template DNA extraction, each isolate was subcultured onto TSA plates for 24 h at 37°C. From the agar plate, 5 colonies were picked and suspended in 100 *μ*L sterile distilled water. Bacterial suspensions were run for 10 min at 94°C [18] in a DNA thermocycler (MultiGene, Labnet International, Inc., USA), and cell debris was removed by centrifugation (12,000 rpm for 1 min). PCR amplification reactions were performed in a volume of 25 *μ*L containing 12.5 *μ*L of KapaTaq 2x Ready Mix (KAPA Biosystems, USA), 20 pmol concentrations of each primer, and 5 *μ*L of DNA template. The cycling parameters [19, 20] were as follows: an initial denaturation at 94°C for 5 min; followed by 26 cycles of 94°C for 1 min, 60°C for 2 min, and 72°C for 3 min; and with a final extension at 72°C for 20 min. The amplified PCR products were subjected to electrophoresis at a 2% agarose gel in 0.5x TBE buffer. 

### 2.3. Phylogenetic Grouping of the Isolates

Phylogenetic classification of *E. coli *isolates was determined using triplex PCR-based phylotyping described by Clermont et al. [21]. Briefly, genomic DNA of bacterial strains was amplified by triplex PCR using primers targeted to three markers, *chuA*, *yjaA*, and TspE4.C2. The phylogenetic grouping was made on the basis of the presence of specific PCR-amplified fragments as follows: group B2 (*chuA*+, *yjaA*+, TspE.C2±), group D (*chuA*−, *yjaA*+, TspE.C2±), group B1 (*chuA*−, *yjaA*±, TspEC2+), and group A (*chuA*−, *yjaA*±, TspE.C2−) (Table 2).

### 2.4. Genotypic Virulence Characterization of the Isolates

Multiplex PCR was used to detect five genes encoding virulence determinants usually associated with extraintestinal pathogenic* E. coli *strains (ExPEC VFs): *neuC *(K1 capsule antigen), *hly *(alpha-hemolysin), *papC *(type P pili), *sfa/foc *(type S pili and type 1C fimbriae), *fimH *(type 1 pili), and *iucC *(aerobactin) [2, 7]. Virulence factor genes were amplified with the primers described in Table 3, in a total volume of 50 *μ*L containing 25 *μ*L of KapaTaq 2x Ready Mix (KAPA Biosystems, USA), 20 pmol concentrations of each primer except *hly* (30 pmol), and 5 *μ*L of DNA template [18]. The reaction conditions were as follows [24]: initial denaturation at 94°C for 4 min followed by 25 cycles of denaturation at 94°C for 30 s, annealing at 63°C for 30 s, and extension at 68°C for 3 min, followed by a final 10 min extension period at 72°C. The amplification products were separated by electrophoresis in a 2% agarose gel containing ethidium bromide. A 100-bp DNA ladder (Kappa Universal) was used in each gel as a molecular size marker. 

### 2.5. Statistical Analysis

The results were analyzed statistically using Chi-square [25]. A *P* value below 0.05 was considered to indicate statistical significance.

## 3. Results 

Sixty-one vaginal *E. coli* isolates from pregnant and nonpregnant women were surveyed for *papG* alleles as predisposing factor to pyelonephritis. Sixty-one fecal isolates from healthy volunteers were also included for comparison. *papG* allele II was the most prevalent allele among both vaginal (32.7%) and fecal (3.2%) isolates, whereas other alleles were found only among vaginal isolates (1.6% for alleles I and III and 3.2% for alleles II + III). Also 90% (9/10) and 78.5% (11/14) of *papG* ^+^ pregnant and nonpregnant women's vaginal isolates were *papG* allele II^+^, respectively (Table 4).

*papG* ^+^ isolates were further genotyped for *E. coli* phylogenetic groups and ExPEC VFs' genes (Table 5). *papG* ^+^ vaginal isolates clustered in groups B2 (78.2%) and D (21.7%), whereas all of the fecal isolates clustered in group D. Except for *sfa/foc*, for all the studied VFs' genes (Table 5), *papG* ^+^ vaginal isolates did not differ significantly in comparison with *papG* ^+^ fecal isolates. Also pregnant and nonpregnant women's vaginal isolates did not differ significantly from each other for the possession of all the studied VFs' genes.

 The vast majority of *papG* allele II^+^ vaginal isolates were clustered in group B2 (81.8%) and much less in group D (18.1%) (Table 6), whereas all of the fecal isolates clustered in group D. Also, most of them were positive for *fimH* (100%),* papC *(100%),* iucC *(90.9%),  and  *hly* (72.7%), and about half of them were positive for *sfa/foc* (45.4%) (Table 6). In addition, the mean of VFs' gene possession was 3.5 (range from 2 to 5).

## 4. Discussion

Here in this work, the vast majority of *papG* allele II^+^ vaginal isolates clustered in group B2 and much less in group D, and most of them were positive for *fimH*, *papC*,* iucC*, and  *hly*, and about half of them were positive for *sfa/foc* (Table 6). Previous studies demonstrated that vaginal *E. coli* share common virulence factor profiles, phylogenetic groups, and serotypes with *E. coli* strains from urinary and neonatal (blood and CSF) origins [11, 20] as the vagina favors colonization by strains that possess features different from those of fecal flora strains, therefore, the vagina can be considered as an anatomical barrier that selects for strains with a greater capacity to cause disease [26]. This high prevalence of phylogenetic group B2 and ExPEC VFs among this work's isolates indicates their pathogenic potential as ExPEC (especially pyelonephritic *E. coli*) since most of UPEC strains belong to phylogenetic group B2 and, to a lesser extent, group D [7]. In addition UPEC strains harbor numerous VFs, such as adhesins (P fimbriae, type 1 fimbriae, S and F1C fimbriae, and afimbrial adhesin), toxins (hemolysin and cytotoxic necrotizing factor), siderophores (the aerobactin system), and polysaccharide coatings (group II capsules) [7, 27, 28].  In comparison with cystitis and fecal isolates, pyelonephritic *E. coli* had a much greater prevalence of phylogenetic group B2, UTI-associated O antigens, and individual VFs, plus higher aggregate VF scores [7]. PapGII and PapC are suggested to be associated with pyelonephritis and that *papG* allele II is one of the significant predictors of this infection [2, 29]. All *papG* allele II^+^ isolates in this work were positive for both *papC* and *fimH* and about half of them were positive for *papC*, *fimH*, and *sfa/foc*. This is consistent with others who demonstrated that type 1, P, S, F1C, and Dr fimbriae are all known to bind to different sites within the human kidney [2] and that P and Type 1 fimbriae appeared to act in synergy to promote colonization of kidney [30]. This possession of multiple fimbrial types contributes to the pathogen's overall success during renal colonization [2]. 

 Pregnant women's isolates did not differ significantly from those of nonpregnant in possession of *papG* allele II (39.1% versus 28.9%), whereas both (32.7%) differed significantly (*P* ≤ 0.05) in comparison with fecal isolates (3.2%) (Table 4). Also *papG* allele II^+^ isolates did not differ significantly from each other regarding the phylogenetic groups and ExPEC VFs' genotypes' distribution (Tables 5 and 6) this indicates that both pregnant and nonpregnant women have the same chance to get pyelonephritis in accordance with this work's proposal, although, previous studies found that acute pyelonephritis is more common in pregnant than in nonpregnant women [11, 31] which means that physiological differences seem to be the critical determinants of predisposition to this infection due to stasis of urine and bacteriuria in the urinary tract caused by relative obstruction [11]. The vaginal ecosystem and especially *Lactobacillus*, as well as intestinal populations of *E. coli*, coitus and the physiological and anatomical conditions of the urinary tract also play a major role in the pathogenesis of urinary tract infections [32]. 

 The possible role of vaginal colonization by such isolates as predisposing factor to pyelonephritis cannot be excluded and required further *in vitro* and *in vivo* analyses, as it previously had been found that vaginal colonization by *E. coli* represents an intermediate stage in extraintestinal *E. coli* pathogenesis [10] and women often suffer from an enhanced susceptibility to recurrent urinary and genital tract infections in association with uropathogenic *E. coli* strains [13]. So, it can be concluded that vaginal colonization by *papG* allele II^+^ *E. coli* is possibly one of the predisposing factors of both pregnant and nonpregnant women to pyelonephritis, but its potential is modified by other factors especially host factors. 

## Figures and Tables

**Table 1:** Nucleotide sequences of PCR primers used to amplify *papG* alleles [19].

Gene	Primer sequence (5′-3′)	Amplicon size (bp)
*papG* allele I		
F	TCGTGCTCAGGTCCGGAATTT	461
R	TGGCATCCCCCAACATTATCG
*papG* allele II		
F	GGGATGAGCGGGCCTTTGAT	190
R	CGGGCCCCCAAGTAACTC
*papG* allele III		
F	GGCCTGCAATGGATTTACCTGG	258
R	CCACCAAATGACCATGCCAGA

**Table 2:** Nucleotide sequences of PCR primers used for phylogenetic grouping of *E. coli* [21].

Gene	Primer sequence (5′-3′)	Amplicon size (bp)
*chuA *		
F	GACGAACCAACGGTCAGGAT	279
R	TGCCGCCAGTACCAAAGACA
*YjaA *		
F	TGAAGTGTCAGGAGACGCTG	211
R	ATGGAGAATGCGTTCCTCAAC
TspE4.C2		
F	GAGTAATGTCGGGGCATTCA	152
R	CGCGCCAACAAAGTATTACG

**Table 3:** Nucleotide sequences of PCR primers used to amplify five ExPEC VFs.

Gene	Primer sequence (5′-3′)	Amplicon size (bp)	Reference
*hly *			
F	AACAAGGATAAGCACTGTTCTGGCT	1,177	[18]
R	ACCATATAAGCGGTCATTCCCGTCA		
*papC *			
F	GACGGCTGTACTGCAGGGTGTGGCG	328	[22]
R	ATATCCTTTCTGCAGGGATGCAATA		
*sfa/foc *			
F	CTCCGGAGAACTGGGTGCATCTTAC	410	[22]
R	CGGAGGAGTAATTACAAACCTGGCA		
*iucC *			
F	AAACCTGGCTTACGCAACTGT	269	[23]
R	ACCCGTCTGCAAATCATGGAT		
*fimH *			
F	TGCAGAACGGATAAGCCGTGG	508	[24]
R	GCAGTCACCTGCCCTCCGGTA		

*hly*: alpha-hemolysin; *papC*: P fimbriae; *sfa/foc*: S fimbriae and F1C fimbriae; *iucC*: aerobactin; and *fimH*: type 1 fimbriae.

**Table 4:** Distribution of *papG* alleles among vaginal and fecal *E. coli* isolates from pregnant and non-pregnant women.

Source of isolates	Study individuals (no.)	No. (%) of isolates positive for *papG* alleles			
		I	II	III	II + III
Vaginal	Pregnant (23)	1 (4.3)	9 (39.1)	0	0
	Nonpregnant (38)	0	11 (28.9)	1 (2.6)	2 (5.2)

Total (%)	(61)	1 (1.6)	20 (32.7)	1 (1.6)	2 (3.2)

Fecal	Pregnant (30)	0	2 (6.6)	0	0
	Nonpregnant (31)	0	0	0	0

Total (%)	(61)	0	2 (3.2)	0	0

**Table 5:** Phylogenetic groups and ExPEC VFs' genes possessed by *papG* ^+^ vaginal and fecal *E. coli* isolates from pregnant and nonpregnant women.

Traits	No. (%) of isolates positive for the indicated trait			
	Vaginal isolates			Fecal isolates
	Pregnant women's isolates (*n* = 10)	Nonpregnant women's isolates (*n* = 14)	Total (*n* = 23)	(*n* = 2)
Phylogenetic groups				
A	0	0	0	0
B1	0	0	0	0
B2	8 (80)	11 (78.5)	18 (78.2)	0
D	2 (20)	3 (21.4)	5 (21.7)	2 (100)
ExPEC VFs' genes				
*fimH *	10 (100)	14 (100)	23 (100)	2 (100)
*papC *	10 (100)	14 (100)	23 (100)	2 (100)
*Sfa/foc *	6 (60)	7 (50.0)	13 (56.5)	0
*hly *	7 (70)	8 (57.1)	15 (65.2)	1 (50)
*iucC *	9 (90)	11 (78.5)	20 (86.9)	2 (100)

**Table 6:** Phylogenetic groups and ExPEC VFs' genes possessed by *papG* allele II^+^ vaginal and fecal *E. coli* isolates from pregnant and nonpregnant women.

Traits	No. (%) of isolates positive for the indicated trait			
	Vaginal isolates			fecal isolates
	Pregnant women's isolates (*n* = 9)	Nonpregnant women's isolates (*n* = 13)	Total (*n* = 22)	(*n* = 2)
Phylogenetic groups				
A	0	0	0	0
B1	0	0	0	0
B2	8 (88.8)	10 (76.9)	18 (81.8)	0
D	1 (11.1)	3 (23.0)	4 (18.1)	2 (100)
ExPEC VFs' genes				
*fimH *	9 (100)	13 (100)	22 (100)	2 (100)
*papC *	9 (100)	13 (100)	22 (100)	2 (100)
*Sfa/foc *	3 (33.3)	7 (53.8)	10 (45.4)	0
*hly *	7 (77.7)	9 (69.2)	16 (72.7)	1 (50)
*iucC *	9 (100)	11 (84.6)	20 (90.9)	2 (100)

## References

[1] Brooks GF, Carroll KC, Butel JS, Morse SA (2007). *Jawetz, Melnick, & Adelberg's Medical Microbiology*.

[2] Lane MC, Mobley HLT (2007). Role of P-fimbrial-mediated adherence in pyelonephritis and persistence of uropathogenic *Escherichia coli* (UPEC) in the mammalian kidney. *Kidney International*.

[3] Marklund B-I, Tennent JM, Garcia E (1992). Horizontal gene transfer of the *Escherichia coli*  
*pap* and *prs* pili operons as a mechanism for the development of tissue-specific adhesive properties. *Molecular Microbiology*.

[4] Stromberg N, Marklund B-I, Lund B (1990). Host-specificity of uropathogenic *Escherichia coli* depends on differences in binding specificity to Gal-1-4Gal-containing isoreceptors. *EMBO Journal*.

[5] Johnson JR, Russo TA, Brown JJ, Stapleton A (1998). *papG* alleles of *Escherichia coli* strains causing first-episode or recurrent acute cystitis in adult women. *Journal of Infectious Diseases*.

[6] Johanson I, Lindstedt R, Svanborg C (1992). Roles of the *pap*- and *prs*-encoded adhesins in *Escherichia coli* adherence to human uroepithelial cells. *Infection and Immunity*.

[7] Johnson JR, Owens K, Gajewski A, Kuskowski MA (2005). Bacterial characteristics in relation to clinical source of *Escherichia coli* isolates from women with acute cystitis or pyelonephritis and uninfected women. *Journal of Clinical Microbiology*.

[8] Kasper DL, Braunwald E, Fauci AS, Hauser SL, Longo DL, Jameson JL (2005). *Harrison's Principles of Internal Medicine*.

[9] Gillespie SH, Hawkey PM (2006). *Principles and Practical Clinical Bacteriology*.

[10] Hooton TM (2000). Pathogenesis of urinary tract infections: an update. *Journal of Antimicrobial Chemotherapy*.

[11] Obata-Yasuoka M, Ba-Thein W, Tsukamoto T, Yoshikawa H, Hayashi H (2002). Vaginal *Escherichia coli* share common virulence factor profiles, serotypes and phylogeny with other extraintestinal *E. coli*. *Microbiology*.

[12] Stapleton AE, Fennell CL, Coder DM, Wobbe CL, Roberts PL, Stamm WE (2002). Precise and rapid assessment of *Escherichia coli* adherence to vaginal epithelial cells by flow cytometry. *Clinical Cytometry*.

[13] Birosoval E, Siegfried L, Kmet'ova M (2004). Detection of virulence factors in *α*-haemolytic *Escherichia coli* strains isolated from various clinical materials. *Clinical Microbiology and Infection*.

[14] Russo TA, Johnson JR (2003). Medical and economic impact of extraintestinal infections due to *Escherichia coli*: focus on an increasingly important endemic problem. *Microbes and Infection*.

[15] Plos K, Connell H, Jodal U (1995). Intestinal carriage of P fimbriated *Escherichia coli* and the susceptibility to urinary tract infection in young children. *Journal of Infectious Diseases*.

[16] MacFaddin JF (2000). *Biochemical Tests for Identification of Medical Bacteria*.

[17] Forbes BA, Sahm DF, Weissfeld AS (2002). *Bailey & Scott's Diagnostic Microbiology*.

[18] Yamamoto S, Terai A, Yuri K, Kurazono H, Takeda Y, Yoshida O (1995). Detection of urovirulence factors in *Escherichia coli* by multiplex polymerase chain reaction. *FEMS Immunology and Medical Microbiology*.

[19] Johnson JR, Brown JJ (1996). A novel multiply primed polymerase chain reaction assay for identification of variant *papG* genes encoding the Gal(*α*1–4)Gal-binding *papG* adhesins of *Escherichia coli*. *Journal of Infectious Diseases*.

[20] Cook SW, Hammill HA, Hull RA (2001). Virulence factors of *Escherichia coli* isolated from female reproductive tract infections and neonatal sepsis. *Infectious Diseases in Obstetrics and Gynecology*.

[21] Clermont O, Bonacorsi S, Bingen E (2000). Rapid and simple determination of the *Escherichia coli* phylogenetic group. *Applied and Environmental Microbiology*.

[22] Le Bouguenec C, Archambaud M, Labigne A (1992). Rapid and specific detection of the *pap*, *afa*, and *sfa* adhesin-encoding operons in uropathogenic *Escherichia coli* strains by polymerase chain reaction. *Journal of Clinical Microbiology*.

[23] Bingen E, Bonacorsi S, Brahimi N, Denamur E, Elion J (1997). Virulence patterns of *Escherichia coli* K1 strains associated with neonatal meningitis. *Journal of Clinical Microbiology*.

[24] Johnson JR, Stell AL (2000). Extended virulence genotypes of *Escherichia coli* strains from patients with urosepsis in relation to phylogeny and host compromise. *Journal of Infectious Diseases*.

[25] Ross SM (2009). *Introduction to Probability and Statistics for Engineers and Scientists*.

[26] Watt S, Lanotte P, Mereghetti L, Moulin-Schouleur M, Picard B, Quentin R (2003). *Escherichia coli* strains from pregnant women and neonates: intraspecies genetic distribution and prevalence of virulence factors. *Journal of Clinical Microbiology*.

[27] Yamamoto S (2007). Molecular epidemiology of uropathogenic *Escherichia coli*. *Journal of Infection and Chemotherapy*.

[28] Tiba MR, Yano T, Leite DDS (2008). Genotypic characterization of virulence factors in *Escherichia coli* strains from patients with cystitis. *Revista do Instituto de Medicina Tropical de Sao Paulo*.

[29] Huang S-H, Wass C, Fu Q, Prasadarao NV, Stins M, Kwang Sik Kim KSK (1995). *Escherichia coli* invasion of brain microvascular endothelial cells *in vitro* and *in vivo*: molecular cloning and characterization of invasion gene *ibe10*. *Infection and Immunity*.

[30] Melican K, Sandoval RM, Kader A (2011). Uropathogenic *Escherichia coli* P and type 1 fimbriae act in synergy in a living host to facilitate renal colonization leading to nephron obstruction. *PLoS Pathogens*.

[31] Ovalle A, Levancini M (2001). Urinary tract infections in pregnancy. *Current Opinion in Urology*.

[32] Andru A (2005). Pathogenesis of urinary tract infections. *Enfermedades Infecciosas y Microbiología Clínica*.

